# Physical activity differences between children from migrant and native origin

**DOI:** 10.1186/1471-2458-14-819

**Published:** 2014-08-09

**Authors:** Wim Labree, Freek Lötters, Dike van de Mheen, Frans Rutten, Ana Rivera Chavarría, Madelon Neve, Gerda Rodenburg, Honorine Machielsen, Gerrit Koopmans, Marleen Foets

**Affiliations:** Institute of Health Policy and Management, Erasmus University, P.O. Box 1738, 3000 DR Rotterdam, The Netherlands; IVO Addiction Research Institute, Heemraadssingel 194, 3021 DM Rotterdam, The Netherlands; Erasmus MC, University Medical Center, P.O. Box 2040, 3000 CA Rotterdam, The Netherlands; Department of Health Promotion, Maastricht University, P.O. Box 616, 6200 MD Maastricht, The Netherlands

**Keywords:** Child health, Transients and migrants, Physical activity, Parenting, The Netherlands

## Abstract

**Background:**

Children from migrant origin are at higher risk for overweight and obesity. As limited physical activity is a key factor in this overweight and obesity risk, in general, the aim of this study is to assess to what degree children from migrant and native Dutch origin differ with regard to levels of physical activity and to determine which home environment aspects contribute to these differences.

**Methods:**

A cross-sectional survey among primary caregivers of primary school children at the age of 8–9 years old (*n* = 1943) from 101 primary schools in two urban areas in The Netherlands. We used bivariate correlation and multivariate regression techniques to examine the relationship between physical and social environment aspects and the child’s level of physical activity. All outcomes were reported by primary caregivers. Outcome measure was the physical activity level of the child. Main independent variables were migrant background, based on country of birth of the parents, and variables in the physical and social home environment which may enhance or restrict physical activity: the availability and the accessibility of toys and equipment, as well as sport club membership (physical environment), and both parental role modeling, and supportive parental policies (social environment). We controlled for age and sex of the child, and for socio-economic status, as indicated by educational level of the parents.

**Results:**

In this sample, physical activity levels were significantly lower in migrant children, as compared to children in the native population. Less physical activity was most often seen in Turkish, Moroccan, and other non-western children (*p* < .05).

**Conclusions:**

Although traditional home characteristics in both the physical, and the social environment are often associated with child’s physical activity, these characteristics provided only modest explanation of the differences in physical activity between migrant and non-migrant children in this study. The question arises whether interventions aimed at overweight and obesity should have to focus on home environmental characteristics with regard to physical activity.

## Background

Globally, the increase of overweight and obesity has reached epidemic proportions [1]. As overweight and obesity lead to numerous chronic diseases, morbidity, quality of life, and mortality are strongly affected both in adults, and in children [2, 3].

Specific symptoms, such as hypertension, hypercholesterolemia, and insulin resistance, which were seen primarily in adults in the last decade, now are becoming more common among children and adolescents [4]. In addition, child overweight affects self-esteem and influences the cognitive and social development of these children [5]. Apart from damaging physical, mental, and social health consequences, the obesity epidemic results in a major economic burden [6]. Furthermore, childhood overweight may develop over time into adolescent obesity and, subsequently, adulthood obesity. Also, parental obesity is an important predictor of obesity in future posterity [7].

Obesity among children is still increasing worldwide and the World Health Organization (WHO) has recognized childhood obesity as one of the most serious public health challenges of the 21^st^ century [8].

It is generally agreed that the etiology of childhood overweight and obesity is complex and multifactorial [9]. Although environmental, genetic, and biological factors play a key role in the energy (im)balance, recent studies show that the increase of overweight and obesity is more likely due to changes in environmental features, referred to as the obesogenic environment, such as changes in physical activity levels and changes in food intake habits [10, 11].

Overweight and obesity are the outcome of an excess of energy intake on energy expenditure, for a longer period of time [12]. In order to design effective prevention programmes, knowledge is needed on modifiable factors in this obesogenic environment, affecting physical activity and nutritional intake [13].

From a systematic review of the European literature, it appeared that migrant children are at higher risk for overweight and obesity than their native counterparts [14]. Often, it is assumed that Body Mass Index (BMI) differences between native and non-native children can be explained by socio-economic position. However, several studies in the United States and Europe showed that migrant or ethnic background remained associated with BMI, independent of socio-economic status [15, 16]. Differences in overweight and obesity between migrant and native children thus require further investigation.

Previous studies have indicated lower levels of physical activity among adult migrant and ethnic groups, as compared to the native population [17]. Limited physical activity may be the result of attitudes regarding the importance of physical activity, as some studies from the United States suggest that in some minority groups physical activity is considered as ‘a waste of time’ or as a ‘luxury’ [18, 19]. Although these attitudes are not subject of the present study, they may influence characteristics related to physical activity in the home environment.

In the present study, we focus on differences between migrant and native children regarding participation in physical activity in the home environment. Physical activity in children is influenced by their physical and social home environment [20]. In the physical environment, both availability, and accessibility of resources are important determinants. More specific: active toys and exercise equipment, such as skip rope or roller skates, which are physical present and within reach, may stimulate physical activity. This also applies to membership of sport clubs. On the other hand, the ease of access to passive toys, such as television or computer, may restrict physical activity [21, 22]. In the social environment, parents play a leading role. The physical activity level they display themselves can be considered as a role model for their children. Besides parental modeling, parental policies are important, in the form of encouraging and prompting children to be physically active or providing transportation to physical activity [23–25].

**Figure 1:**
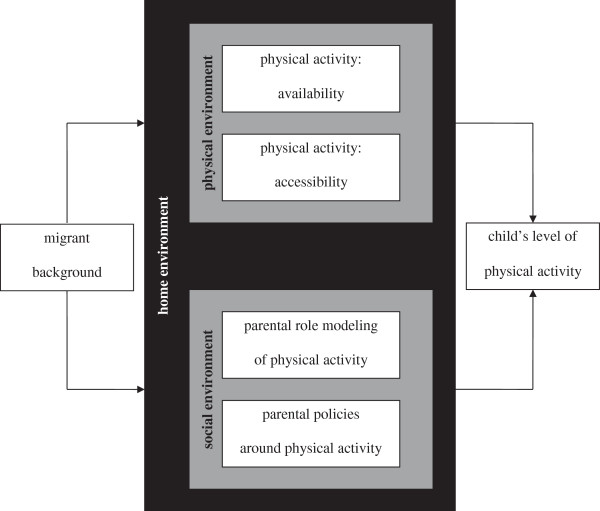
**Conceptual model for the influence of the home environment on physical activity; based on Gattshall and colleagues [** [26] **].**

In this study, we focus on differences in the levels of physical activity between migrant and native Dutch children and on the role of the physical and social home environment in these differences. In Figure 1, the conceptual model of this study is presented. Migrant background is not considered as a factor that directly can explain possible physical activity differences. We hypothesize that potential differences between migrant and native children can be explained to some degree by differences in the home environment.

Socio-economic position may also influence the physical activity level of the child [27]. Because, in general, the socio-economic status of migrants is lower than that of the native population, we will adjust for the parental socio-economic position. Finally, we will take into account age and sex of the child, because these variables affect physical activity levels [28].

### Study aim

The first aim of this study is to compare the level of physical activity between children from migrant and native origin in The Netherlands. The second study aim is to investigate to what degree differences in the physical activity level between these children can be explained by differences in their physical and social home environments.

## Methods

### Design study

To achieve our research aim, we performed a cross-sectional study, as part of the longitudinal IVO Nutrition and Physical Activity Child CohorT (INPACT) study. The INPACT study is a shared research project conducted by the IVO Addiction Research Institute, and the Institute of Health Policy and Management, a department of the Erasmus University Rotterdam, with approval of the Medical Ethics Committee of the Erasmus MC, University Medical Center Rotterdam.

This four year observational study focuses on modifiable factors affecting overweight and obesity in Dutch primary school children aged 8–9 years to 11–12 years. The present study was based on the first wave of data collection in the school year of 2008/2009.

### Study population

We approached parents of primary school children from 8–9 years old. Collaborating primary schools in this project were located in two cities and adjacent areas in The Netherlands: Rotterdam and Eindhoven. Many children from migrant and non-migrant origin attend school and live in one of these cities.

In a letter, in which our research goals were explained, schools were invited for participation in the INPACT study. Schools were excluded if they were participating in prevention activities, at the time of the study, as this could influence the measures. A total of 101 schools took part in the study. Parents received an information letter. Out of 3162 parent–child dyads, 1943 (61.5%) dyads decided to contribute to this study. Subjects at baseline were children in group 5 of Dutch primary schools (8–9 years old). Participation was on the basis of written informed consent by the parent.

All information was derived from questionnaires. The primary caregiver of the child, mostly the mother, was asked to fill out the questionnaire. Therefore, measures were proxy-reported outcomes. In order to stimulate participation among the two largest migrant groups, parents from Turkish and Moroccan origin, received a letter in their native language. Also, they could ask for assistance in this language by interpreters, while filling out the questionnaire.

### Measurements

The general part of the questionnaire included questions on the child’s age, sex, migrant background, and socio-economic position.

Children were considered as having a migrant background, when at least one of their parents was born outside The Netherlands, which is in accordance with current Dutch practice. If both parents were born in different foreign countries, the maternal country of birth was used to define the child’s country of origin [29].

Based on this current Dutch practice, we have distinguished five groups in our sample: native Dutch children (*n* = 1546), children with a Turkish background (*n* = 93), children with a Moroccan background (*n* = 66), and two additional groups, containing children from a variety of countries, one with children from non-western origin (*n* = 105) and one with children from western origin (*n* = 133).

Socio-economic position was determined by the educational level of the parent that achieved the highest level, classified into categories: low (primary school, lower vocational education, general education), middle (secondary school, intermediate vocational school), or high (higher vocational school, university).

#### Outcome measure: child’s physical activity

To assess the children’s physical activity level, we used a questionnaire, to be filled out by the primary caregiver of the child. This instrument has been developed by the National Institute for Public Health and the Environment and by the local Public Health Services, based on previous studies in other countries [30]. This assessment followed suggestions of Welk et al. [31], who advised to assess various types of physical activity in specific key times and places, in order to enhance the validity. We asked how often, based on a normal week, children [1] went to school by foot or by bicycle (active transport), [2] played inside or outside, and [3] participated in a sport or at a sport club. Physical activity was asked in terms of duration (minutes) and of frequency (times per week).

Based on the questions, we calculated the amount of minutes that the child spends on physical activity per week and divided the total number of minutes by 7 to assess the mean number of minutes of physical activity per day (mean = 64.7; SD = 21.2).

#### Physical environment: availability and accessibility

Availability of resources in the home environment (physical presence) was assessed by means of a checklist with 14 items that referred to availability of active toys and exercise equipment, which could be scored by presence (score “1”) or absence (score “0”) of these aspects. This list was based on a measuring instrument developed by Gattshall and colleagues [26] and was adapted for application in The Netherlands.

Similarly, the primary caregiver had to score whether the child joined 16 types of specific sport clubs (e.g., tennis, soccer). A sum score, ranging from 0 to 14 for toys and equipment (mean = 6.5; SD = 2.1) and ranging from 0 to 16 for sport clubs (mean = 1.3; SD = 0.7), was calculated for analysis.

The accessibility or the possibility in the home environment (ease of access) regarding toys and equipment were assessed by 3 questions concerning active toys (e.g., skip rope, rollerskates) and 4 questions concerning passive toys (e.g., television, computer). Respondents could answer on a 5-point Likert scale. Each item scale ranged from 1 (never within reach) to 5 (always within reach). Regarding active toys, the mean accessibility score was 12.9 (SD = 1.8; range = 3–15) and regarding passive toys, this score was 15.0 (SD = 2.9; range = 5–20). Reliability and internal consistency of this instrument were high. Cronbach’s alpha for the accessibility total score is .81 for active toys and .77 for passive toys.

#### Social environment: parental role modeling and parental policies

Physical activity of the primary caregiver was used as an indicator of parental role modeling and was measured by the Short QUestionnaire to ASsess Health-enhancing physical activity (SQUASH) by Wendel-Vos and colleagues [32]. This instrument consists of various physical activities, of which the number of reported days, the duration (minutes), and the level (light activity, moderate activity, and vigorous activity) were assessed.

At the end, we calculated the amount of minutes that the primary caregiver spends on moderate and vigorous activities per week and divided the total number of minutes physical activity by 7 to assess the mean number of minutes of physical activity per day (mean = 65.0; SD = 22.8).

Parental policies to support the child’s physical activity were assessed by survey questions developed by Gattshall and colleagues [26]. These policies, as a part of the social environment, consisted of 5 questions, using a 5-point Likert scale (see Appendix). The total scale ranged from 1 (always supportive) to 5 (never supportive). For descriptive purposes, we distinguished between two groups, based on the data distribution: lower scores suggesting more supportive policies (47%) and higher scores suggesting less supportive policies (53%). Cronbach’s alpha for the parental policies total score is .64, which can be considered as moderate.

### Analysis

The characteristics of the study population have been calculated for each group. To compare the mean level of physical activity between migrant and native Dutch children, differences were analysed with an ANOVA. Our reference group consisted of native Dutch children. Additionally, to compare other group differences, we analysed scores using a *t*-test, an ANOVA or a chi-square test.

In accordance with our conceptual model, bivariate correlations were performed to determine the relationships between all independent variables (age, sex, educational level, home environmental characteristics) and the dependent variable (child’s level of physical activity).

Subsequently, we used multivariate linear regression to investigate whether the relation between migrant background and level of child’s physical activity could be explained by aspects in the physical and social home environment. Because age, sex, and educational level have an independent effect on the child’s physical activity level, we have controlled for these variables in the analysis. Three models were tested. In the first model, we adjusted for age, sex, migrant background, and the parental educational level. In the second model the resources in the physical environment were included: both the availability of active toys and equipment, sport club membership, and the accessibility of active and passive toys. In the final model (model 3), the features in the social environment were included: parental role modeling and supportive parental policies. Missing values were excluded from the analyses. Data were analysed using the SPSS program (version 19.0).

## Results

Sample characteristics of all children (*n* = 1943) are presented in Table 1. The first part of this table shows the mean age of the children, the percentages of boys and girls, and parental educational level, in each group. The educational level of the parents of the native Dutch children and the other western children is higher than that of the Turkish, Moroccan, and the other non-western children.

**Table 1:** **Sample characteristics**

	Dutch	Turkish	Moroccan	non-western	western
	(***n*** = 1546)	(***n*** = 93)	(***n*** = 66)	(***n*** = 105)	(***n*** = 133)
Age, M (SD) [missing]	8.2 (0.45) [4]	8.6 (0.67) [1]	8.5 (0.61) [1]	8.4 (0.63) [3]	8.3 (0.52) [2]
Boys, *n* (%)	778 (50.3)	38 (40.9)	36 (54.5)	44 (41.9)	74 (55.6)
Girls, *n* (%)	768 (49.7)	55 (59.1)	30 (45.5)	61 (58.1)	59 (44.4)
Educational					
level parents,%					
- Low	12.5	42.4^*^	34.5^*^	26.4^*^	13.9^*^
- Medium	39.7	37.6^*^	37.9^*^	35.2^*^	32.8^*^
- High	47.9	20.0^*^	27.6^*^	38.5^*^	53.3^*^
[missing]	[23]	[8]	[8]	[14]	[11]
Availability (toys), M (SD) [missing]	6.9 (1.8) [5]	3.9 (2.1)^*^ [3]	4.3 (2.2)^*^ [3]	5.6 (2.5)^*^ [9]	5.8 (2.2)^*^ [7]
Availability (sport clubs), M (SD) [missing]	1.4 (0.6) [86]	1.2 (0.8)^*^ [4]	1.0 (0.7)^*^ [9]	1.1 (0.8)^*^ [13]	1.3 (0.9)^*^ [11]
Accessibility (active), M (SD) [missing]	13.1 (1.6) [14]	10.8 (2.1)^*^ [3]	12.4 (2.4)^*^ [4]	12.2 (2.0)^*^ [11]	12.8 (1.9)^*^ [9]
Accessibility (passive), M (SD) [missing]	14.9 (2.9) [57]	15.6 (3.0) [13]	15.8 (3.2) [14]	15.6 (2.9) [12]	14.8 (2.9) [10]
Role modeling, M (SD) [missing]	64.6 (22.5) [190]	59.1 (24.6)^*^ [28]	70.6 (25.8)^*^ [24]	73.7 (25.5)^*^ [24]	65.1 (22.9)^*^ [65]
Supportive parental policies,% [missing]	47.0 [4]	40.2^*^ [1]	45.3^*^ [2]	46.5^*^ [6]	52.8^*^ [6]
Child’s physical activity level, minutes M (SD) [missing]	67.9 (21.0) [8]	38.0 (18.7)^*^ [2]	56.9 (26.8)^*^ [4]	41.1 (18.7)^*^ [3]	62.0 (21.4)^*^ [3]

^*^ *p* < .05.

The mean number of minutes of physical activity per day, is displayed in Table 1. Overall, mean scores differed between the groups of children (*p* < .05). Dutch children had the highest physical activity score (mean = 67.9; SD = 21.0). All migrant children had lower physical activity scores. Turkish children displayed the lowest scores, followed by other non-western and Moroccan children.

Availability of active toys and equipment and membership of sport clubs was highest among native children, compared to migrant children. Scores between the groups of children differed significantly (*p* < .05). Furthermore, children from native Dutch and other western origin did show higher scores regarding accessibility to active toys (significant) and lower scores regarding accessibility to passive toys (not significant).

With regard to parental role modeling, as expressed in the mean number of minutes of physical activity per day, only migrant parents from Turkish origin show lower means. Both other western and other non-western, and Moroccan parents have higher means, even compared to native Dutch parents (mean = 64.6; SD = 22.5). Regarding parental policies, parents of Turkish, Moroccan, and non-western children are less supportive (40.2%, 45.3%, and 46.5%). Parents of western children show even more supportive parental styles than native Dutch parents (52.8%).

Table 2 presents bivariate correlations (pearson and spearman’s correlation coefficients, and chi-square values) between all independent variables and the dependent variable. Apart from sex, positive relationships are found with regard to availability (toys), availability (sport clubs), accessibility (active), role modeling, and supportive parental policies.

**Table 2:** **Bivariate correlations**

	Child’s physical activity level		
Age, pearson correlation coefficient	−.005 (*p* = .830)		
Sex, *t* value	5.829^*^ (*p* = .000)	Boys, M (SD)	73.8 (22.9)
		Girls, M (SD)	55.9 (19.1)
Educational level parents, spearman’s rho	.004 (*p* = .521)	Low, M (SD)	62.8 (23.2)
		Middle, M (SD)	61.6 (20.9)
		High, M (SD)	63.4 (20.5)
Availability (toys), pearson correlation coefficient	.084^*^ (*p* = .001)		
Availability (sport clubs), pearson correlation coefficient	.280^*^ (*p* = .000)		
Accessibility (active), pearson correlation coefficient	.059^*^ (*p* = .046)		
Accessibility (passive), pearson correlation coefficient	.001 (*p* = .961)		
Role modeling, pearson correlation coefficient	.122^*^ (*p* = .000)		
Supportive parental policies, chi-square value	88.845^*^ (*p* = .000)	Non-supportive, M (SD)	56.3 (19.7)
		Supportive, M (SD)	74.9 (22.4)

^*^ *p* < .05.

In the multivariate regression analyses, taking into account age, sex, and migrant background, Turkish, Moroccan, and other non-western children showed significantly lower physical activity scores than native children (see Table 3). Non-native children with a western background did not differ from native Dutch children. Age was not associated with physical activity; sex was associated. We did not find a significant association between educational level of the parents and physical activity level. The total explained variance is 3% in the first model.

**Table 3:** **Predictors of child’s physical activity: results of multivariate regression analyses**

Variables	Model 1			Model 2			Model 3		
	β	t	***p***	β	t	***p***	β	t	***p***
Age	0.02	0.67	0.08	0.02	0.85	0.40	0.02	0.57	0.57
Sex girl	−0.11^*^	−3.80	0.00^*^	−0.17^*^	−6.23	0.00^*^	−0.17^*^	−6.00	0.00^*^
Background									
- Turkish	−0.11^*^	−3.84	0.00^*^	−0.12^*^	−4.21	0.00^*^	−0.12^*^	−4.24	0.00^*^
- Moroccan	−0.07^*^	−2.44	0.02^*^	−0.05^*^	−1.80	0.04^*^	−0.05^*^	−1.87	0.04^*^
- Non-western	−0.05^*^	−1.85	0.04^*^	−0.04^*^	−1.57	0.05^*^	−0.04^*^	−1.56	0.05^*^
- Western	−0.02	−0.71	0.48	−0.02	−0.72	0.47	−0.02	−0.84	0.39
Educational level									
- Middle	0.02	0.49	0.63	0.01	0.29	0.77	0.00	0.09	0.93
- High	0.03	0.62	0.54	0.02	0.62	0.53	0.00	0.01	0.99
Availability (toys)				0.00	0.15	0.88	0.00	0.01	0.10
Availability (sport clubs)				0.33^*^	11.80	0.00^*^	0.33^*^	11.78	0.00^*^
Accessibility (active)				0.05^*^	1.82	0.05^*^	0.05^*^	1.75	0.05^*^
Accessibility (passive)				0.04	1.46	0.15	0.04	1.47	0.14
Role modeling							0.07^*^	2.74	0.00^*^
Supportive parental policies							0.10^*^	3.73	0.00^*^
adjusted R^2^	0.03			0.13			0.15		
R^2^ change	0.03^*^			0.10^*^			0.02^*^		

^*^ *p* < .05.

When adding the indicators of the physical environment to our model (second model), the previously observed ethnic differences hardly changed. Membership of sport clubs was related to the child’s physical activity level. Furthermore, only access to active toys was associated. The relationships between ethnicity, the other independent variables, and the dependent variable remained the same. The total explained variance increased (13%).

Finally, ethnic differences, expressed as β, did not change when indicators of the social environment were added (third model). Role modeling and supportive parental policies were significantly related to the child’s physical activity level. The contribution of all other variables did not change. The total explained variance is 15%.

## Discussion and conclusions

Results from this study show that physical activity levels in children were significantly lower among migrant children, as compared to children in the native population. Especially, Turkish children show a very low level of physical activity.

Our results are difficult to compare with previous studies conducted in the United States and in Europe, for example, England, because the migrant groups in these studies cannot be compared with the specific migrant groups in our study [33–35]. However, a recent Dutch study among preschool children showed similar results with our findings, at least to some degree [36]. In this study, children with a non-western migrant background played less often outside, as compared to their native counterparts. Unfortunately, non-western migrant groups were not further specified. Similar results are seen in a Swiss study, which aimed to assess physical activity in preschool children from different multicultural backgrounds [37]. Migrant children showed more sedentary behavior, as compared to children in the native population, although no specific migrants groups were distinguished in this study. To our knowledge, our study is the first that also aimed to explain differences in physical activity levels between migrant and native primary school children. This also appears from recent reviews on determinants of physical activity and sedentary behavior in young people [38, 39]. In these reviews, few studies were selected that included ethnicity as a determinant of physical activity. However, none of the examined studies concentrated on explaining these ethnic differences.

Although the clear differences in levels of physical activity in our study between children from migrant and native Dutch origin, traditional physical and social characteristics of the home environment provided only modest explanation of these differences. Also, we did not find a significant association between parental education and physical activity level. In the earlier mentioned study by van Rossem and colleagues [36] this was also the case: no physical activity differences were found, according to the mother’s educational level. Nevertheless, some of the included determinants, such as membership of sport clubs, accessibility of active toys and equipment (physical environment), and role modeling and supportive parental policies (social environment), did contribute to the explained variance in physical activity levels in our sample. However, the explained variance remained modest.

Unfortunately, we can only speculate why we were not successful in explaining the lower levels of physical activity, especially among Turkish, Moroccan, and other non-western children, although our point of departure was an explanatory model based on existing literature. The independent variables included in our study were all based on validated instruments.

We did only include the availability of active toys, and not the availability of passive equipment such as televisions, and (game) computers. Although a question was included in the survey, also more active game computers, such as the Wii, were included, and it was not possible to distinguish between these computer systems. However, access to this passive equipment did not contribute to differences in activity levels.

Contrary to our expectation, the physical activity level of Moroccan, other non-western, and other western parents was higher than that of the native Dutch parents. Therefore, it could only contribute to the explanation of the relatively lower levels of physical activity among Turkish children. We assessed the physical activity level of the primary caregiver, which in most cases was the mother. Assessing the physical activity level of the father might be more adequate as an indicator of role modeling for boys.

In this study, we assessed physical activity of the children by means of parental reports. We did not use self-reports by the children because they have more difficulty with cognitive tasks of recalling at this age than adults [40]. Due to concerns of expense, this method is most often used in observational studies. More direct methods such as observations might be more valid. No information is available on ethnic differences in proxy reports on physical activity levels of children. Concerning adults, our physical activity questionnaire is considered to be a valid measure [41]. Nevertheless, we recommend further evaluation of the validity of parental reports.

Furthermore, we assessed socio-economic position, although it is a multidimensional concept, only by educational level. A recent study showed that the relationship between socio-economic status and ethnic differences in health can differ, according to which indicators are applied [42]. Educational level as main indicator was chosen because most parents in this study are first generation migrants. These migrants relatively often experience little formal education [43]. Educational level determines the level of knowledge on healthy exercise. Although income level also affects the physical activity level, as low income levels might make it more difficult to participate in sport clubs, the amount of missing values on the income level of the families was enormous.

Also, we assessed migrant origin by the country of birth of the parents, because it has the advantage to be objective and stable [44]. Despite the classification into two well-defined groups, children with a Turkish and a Moroccan background, the classification of other non-native children into two heterogeneous groups, other western and other non-western children, was necessary, because of the limited number of children within the separate groups, but subject to critique. Nevertheless, our findings suggest that, concerning physical activity level, western children resemble more the native Dutch, whereas the non-western children resemble more the Turkish and Moroccan children.

It is important to point out that our study has a cross-sectional design. Therefore, we can only describe differences between groups at a single point of time. Causal conclusions cannot be drawn from this study.

Concluding, we did find differences in physical activity levels between children from migrant and native Dutch origin. However, our hypothetical model provided a modest explanation of these differences. Therefore, the question arises whether interventions aimed at reducing overweight and obesity should have to focus on traditional home characteristics with regard to participation in physical activity. Instead, we suggest further exploring parental attitudes regarding the importance of physical activity for children from migrant origin, since other studies found that some minority groups hold negative attitudes toward physical activity [18, 19]. Furthermore, physical activity is an important determinant of BMI, as are sedentary habits. We also recommend that future studies should investigate differences in sedentary behavior among primary school children from migrant and native Dutch origin.

## Appendix: Physical activity at the home environment: example questionnaire items

1. physical activity: availability
  • an inventory of active toys and exercise equipments (e.g., skip rope, rollerskates), as well as a list of sportclubs (e.g., tennis, soccer)
2. physical activity: accessibility
  • Are these toys and equipments stored out of sight of the child?
3. parental role modeling of physical activity
  • How many times a week you visit a sport club?
4. parental policies around physical activity
  • How often do you encourage your child to be physically active?
